# Supplementary material for: Androgen regulation of pulmonary AR, TMPRSS2 and ACE2 with implications for sex-discordant COVID-19 outcomes
Source: Sci Rep. 2021 May 27;11:11130. doi: 10.1038/s41598-021-90491-1 (PMC8159988; doi:10.1038/s41598-021-90491-1)

**Supplementary Materials**

**Androgen regulation of pulmonary AR, TMPRSS2 and ACE2 with implications for sex-discordant COVID-19 outcomes**

Mehdi Baratchian^1^, Jeffrey M. McManus^1^, Mike P. Berk^1^, Fumihiko Nakamura^1^, Sanjay Mukhopadhyay^2^, Weiling Xu^3,4^, Serpil Erzurum^3,4^, Judy Drazba^5^, John Peterson^5^, Eric A. Klein^6^, Ben Gaston^7^ and Nima Sharifi^1,6,8,*^

^1^ Genitourinary Malignancies Research Center, Lerner Research Institute, Cleveland Clinic

^2^ Pathology and Laboratory Medicine Institute, Cleveland Clinic

^3^ Department of Pathobiology, Lerner Research Institute, Cleveland Clinic

^4^ Respiratory Institute, Cleveland Clinic

^5^ Imaging Core, Lerner Research Institute, Cleveland Clinic

^6^ Department of Urology, Glickman Urological and Kidney Institute, Cleveland Clinic

^7^ Herman Wells Center for Pediatric Research, Indiana University School of Medicine

^8^ Department of Hematology and Oncology, Taussig Cancer Institute, Cleveland Clinic


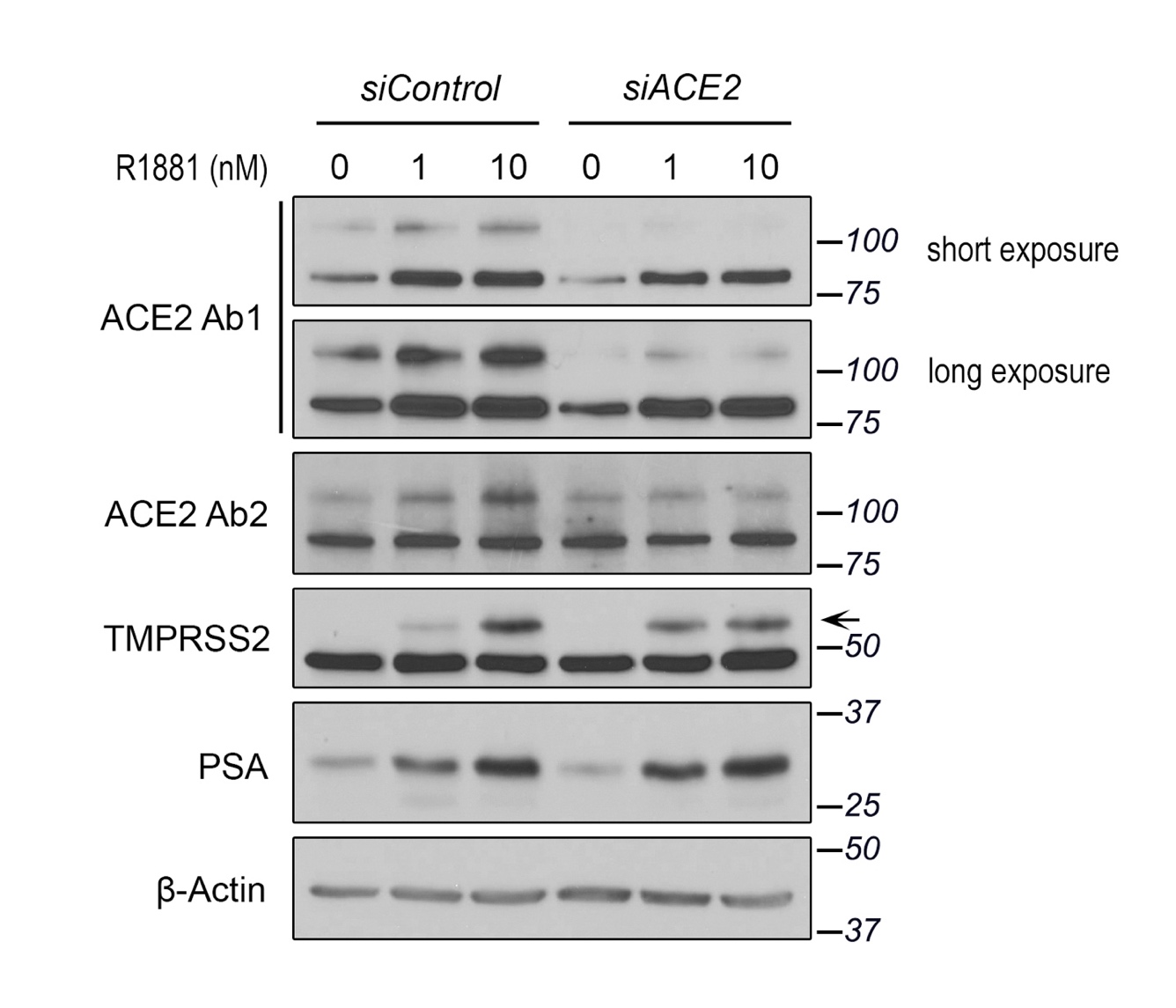


**Figure S1. Validation of the specificity of the anti-ACE2 antibodies used in this study.** LNCaP cells were transfected with control or ACE2-targeting siRNA pool (ON-TARGET SMARTpool, Cat#L-005755) at a final concentration of 20nM. Twenty-four hours later, each group was treated with vehicle (ethanol), or 1, or 10nM of R1881 to activate AR signalling. Whole cell lysates were extracted 72 hours post-treatment and analyzed for the expression of ACE2 using two distinct antibodies (Ab1: ab15348, Ab2: CST#4533). TMPRSS2 and PSA levels served as positive controls for AR induction. Arrow indicates the location of TMPRSS2-specific band.


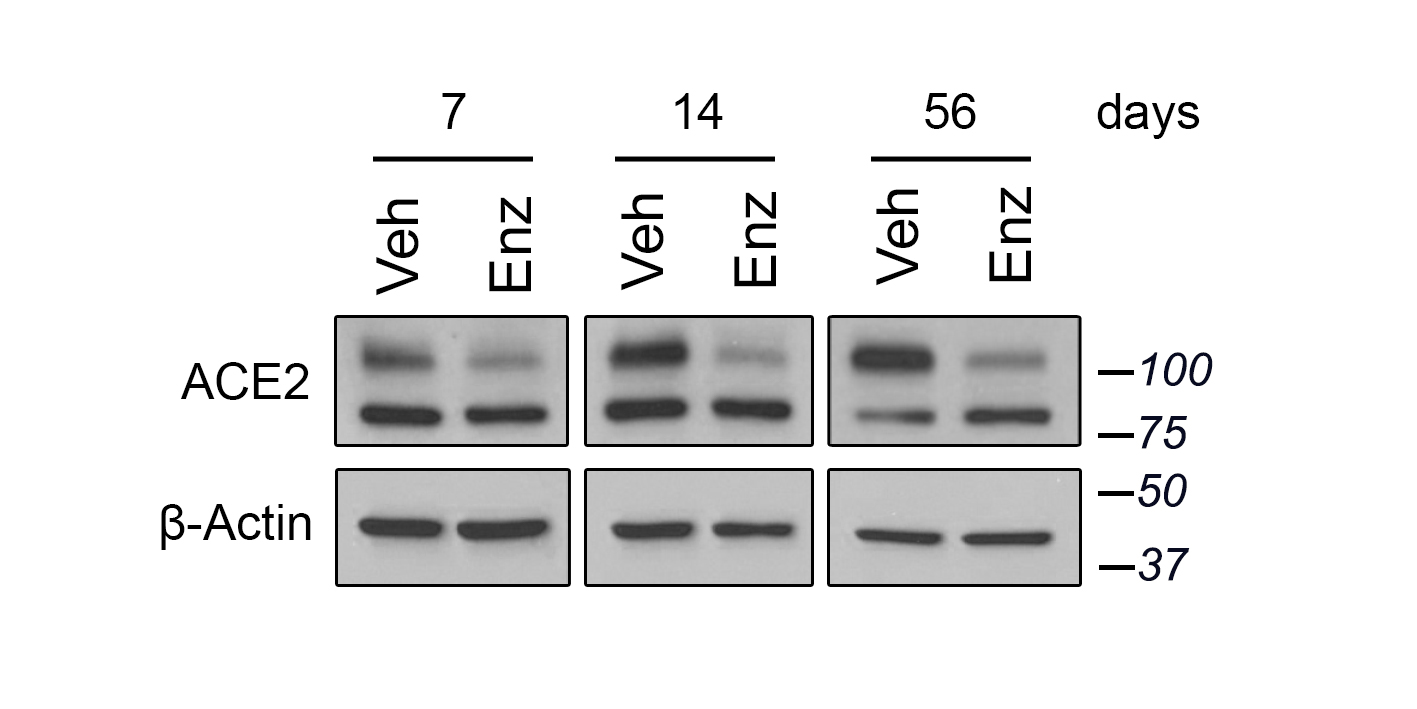


**Figure S2. Enzalutamide strongly suppresses the expression of ACE2 in a prostate cancer cell line model.** Immunoblots showing the expression levels of the ACE2 protein in LNCaP cells treated with vehicle (DMSO) or Enz (10 µM) for 7, 14 or 56 days. The immunoblots are representative of two biological repeats.

**
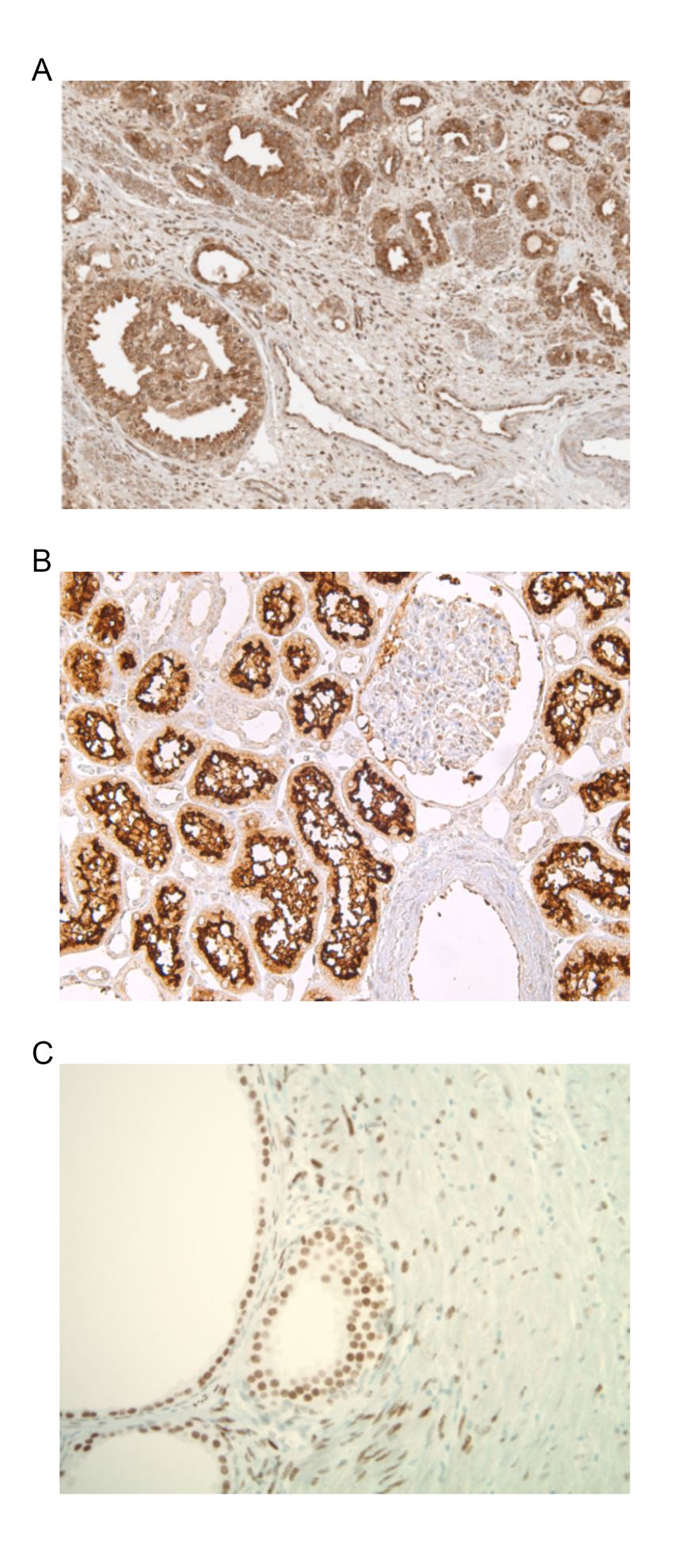
**

**Figure S3. Optimization of the IHC methods for TMPRSS2, ACE2 and AR analyses on positive control tissues. (**A) TMPRSS2 IHC on human prostate tissues. Paraffin-embedded formalin-fixed human prostate tissues were sectioned 5 μm thick. Antigen retrieval was performed using a tris/borate/EDTA buffer (Discovery CC1, 06414575001; Roche), pH 8.0 to 8.5. Anti-TMPRSS2 polyclonal antibody (1:3000, RT, 1h, cat# ab92323, Abcam) was used to detect TMPRSS2 expression in tissue samples for analyses. TMPRSS2 expression in human prostate served as positive control, while no primary with only secondary antibody alone was performed for negative control. (B) ACE2 IHC on human kidney tissues. Paraffin-embedded formalin-fixed kidney tissues were sectioned at 5 μm. Goat anti-ACE2 polyclonal antibody (1:400, RT, 1h, cat# AF933, R&D Systems) was used for analyses after antigen retrieval with EDTA, pH 9 for 32 min. ACE2 expressed in proximal tubules of human kidney served as positive control, while negative control of secondary antibody alone was performed. (C) AR IHC on human prostate tissues. Immunohistochemical stains were performed on formalin-fixed, paraffin-embedded prostate tissues cut in 4µM sections on the Ventana Benchmark Ultra automated immunostainer (Ventana Medical Systems (VMS), Tucson AZ).  Online deparaffinization was followed by online epitope retrieval using a high pH tris based solution (VMS Ultra CC1) for 64 minutes at 100°C.  The slides were incubated with the anti-AR primary antibody (mouse monoclonal AR441, Dako (Agilent), Santa Clara, CA) at 1:100 dilution for 32 minutes with no heat. Localization of the antigen-antibody complex was achieved using the VMS OptiView DAB detection kit.  Control tissue is prostate where carcinoma will be immunoreactive and stromal and lymphoid cells will be non-immunoreactive. The slides were counterstained with hematoxylin and the images were captures at 400x magnification.

**
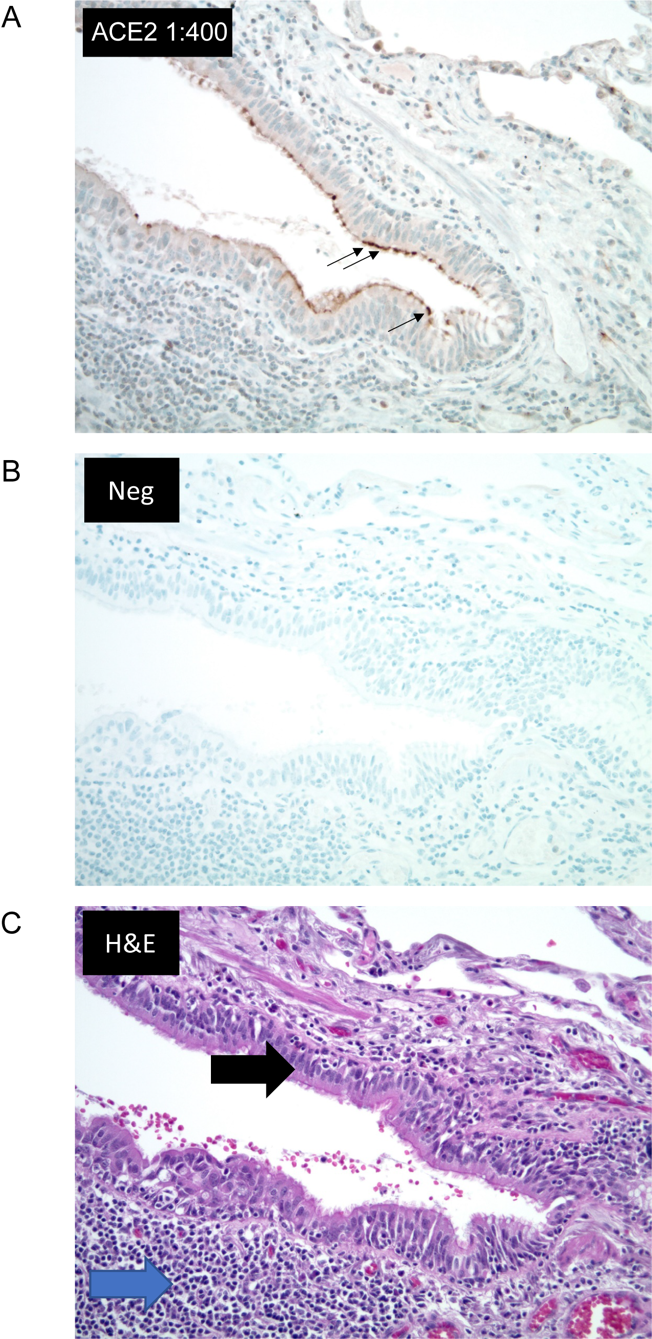
**

**Figure S4. Demonstration of the specific ACE2 staining in a representative human male lung.** A) Staining with ACE2 Ab at 1:400 dilution: Apical membrane staining in ciliated columnar cells (arrows). B) Staining with secondary only Ab served as negative (Neg) control; no staining in any cell type was detected. C) Haematoxylin and eosin (H&E) staining: bronchiole from lung tissue lined by ciliated columnar cells (black arrow). Lymphocytes are indicated by blue arrow. The images were captured at 400x magnification.

**Table S1. Clinical characteristics of patients whose lung samples were used for immunohistochemical analyses.**

| Case number | Sex | Age | BMI (kg/m2) | History | Comorbidities |
| --- | --- | --- | --- | --- | --- |
| 1 | Male | 75 | 32.68 | 44 PY, ex-smoker quit 2018, wedge resection for LVRS | Lung cancer, CHF, type 2 diabetes |
| 2 | Male | 68 | 28.54 | 57 PY, ex-smoker, quit 2007, lobectomy for adenocarcinoma | Lung cancer, COPD, hypertension, prostate cancer |
| 3 | Male | 53 | 28.51 | PY N/A, ex-smoker, quit 2011, transplant for emphysema | COPD, prior MAI, CAD, asthma, hypertension |
| 4 | Male | 68 | 26.53 | 67.5 PY (1.5 PPD, 45Y), ex-smoker, quit 2006, transplant for emphysema | Severe COPD |
| 5 | Male | 60 | 25.84 | 40-60 PY, ex-smoker quit July 2018, wedge resection for LVRS | Severe COPD |
| 6 | Female | 51 | 33.85 | 15-20 PY, recent cessation (1 month), lobectomy for adeno | Lung cancer, asthma, hypothyroidism |
| 7 | Female | 79 | 28.84 | 60 PY, continues to smoke, lobectomy for adenocarcinoma, has emphysema | Lung cancer, GERD |
| 8 | Female | 60 | 16.21 | 41 PY, current smoker (tried to quit Jan 2020), lobectomy for necrotizing granuloma, has emphysema | Multiple sclerosis, SLE, mycobacterial lung granuloma |
| 9 | Female | 59 | 29.92 | 25 PY smoker, quit in 2003 (16 years prior), lobectomy for adenocarcinoma, RB in background | Lung cancer, hyperlipidemia, hypothyroidism |
| 10 | Female | 79 | 26.3 | Former smoker (quit 8y prior), 1 pack a week for 55 years, wedge resection for intraparench LNs | Thymoma, hypertension, hyperlipidemia |
| 11 | Female | 65 | 35.56 | smoker, 20-30 PY with intermittent cessation, stopped 2 years ago | Breast cancer, lung cancer, arthritis |
| 12 | Male | 17 | 16.24 | never-smoker | Pneumothorax |
| 13 | Male | 66 | 26.92 | never-smoker | Interstitial lung disease, type 2 diabetes, chronic kidney disease |
| 14 | Male | 80 | 26.54 | never-smoker | Lung cancer, type 2 diabetes, hyperlipidemia, chronic kidney disease |
| 15 | Male | 81 | 21.97 | never-smoker | Lung cancer, CAD, hyperlipidemia |
| 16 | Male | N/A | N/A | never-smoker | N/A |
| 17 | Female | 39 | 22.36 | never-smoker | Lung cancer, sarcoidosis, polycystic ovaries |
| 18 | Female | 53 | 45.04 | never-smoker | Lung cancer, breast cancer, hypertension |
| 19 | Female | 30 | 25.1 | never-smoker | Pulmonary sequestration |
| 20 | Female | 63 | 25.54 | never-smoker | Carcinoid tumor of lung, mild intermittent asthma |

CAD: coronary artery disease, CHF: congestive heart failure, COPD: chronic obstructive pulmonary disease, GERD: gastroesophageal reflux disease, LN: lymph node, LVRS: lung volume reduction surgery, MAI: Mycobacterium avium-intracellulare, PY: pack-year, RB: respiratory bronchiolitis, SLE: systemic lupus erythematosus, N/A: not available.


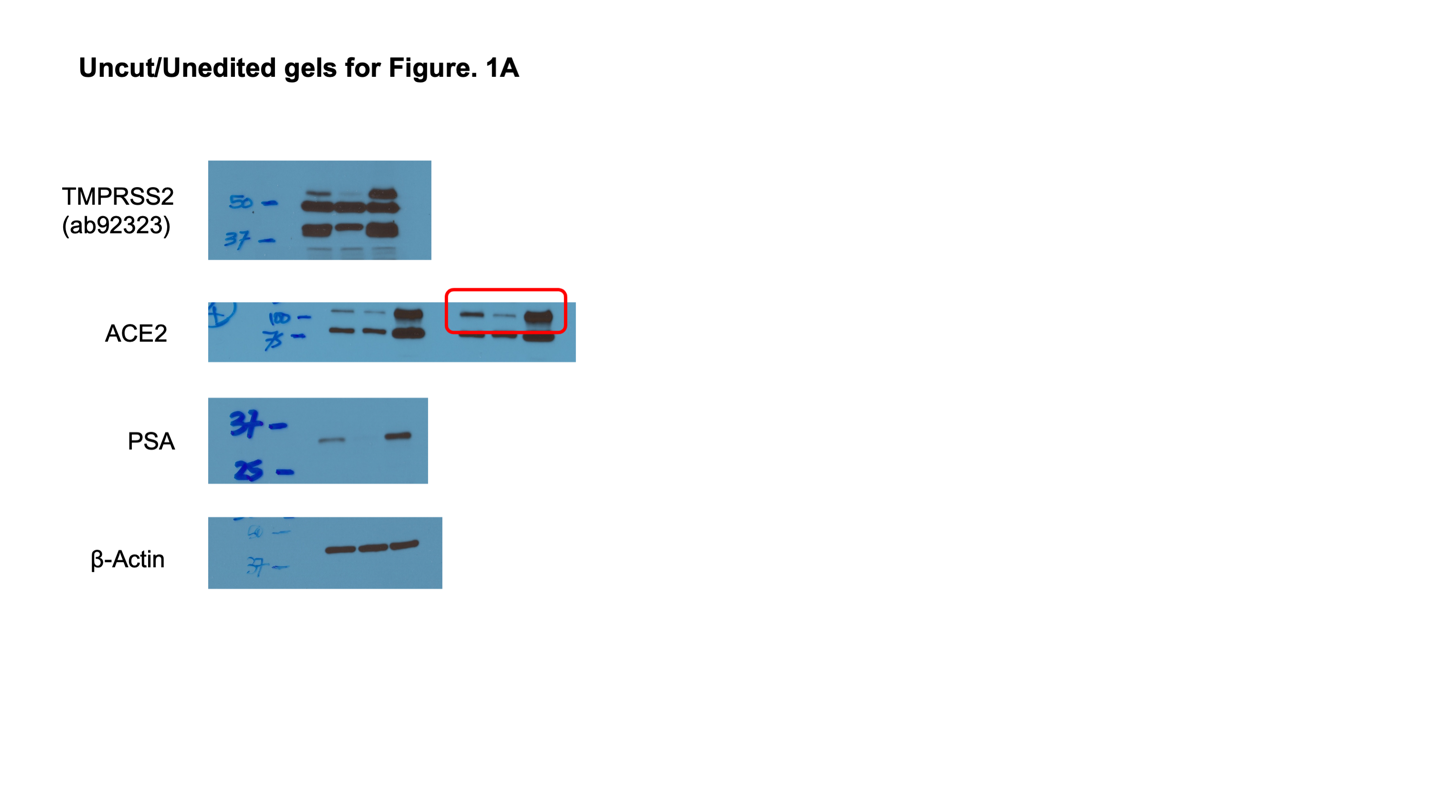

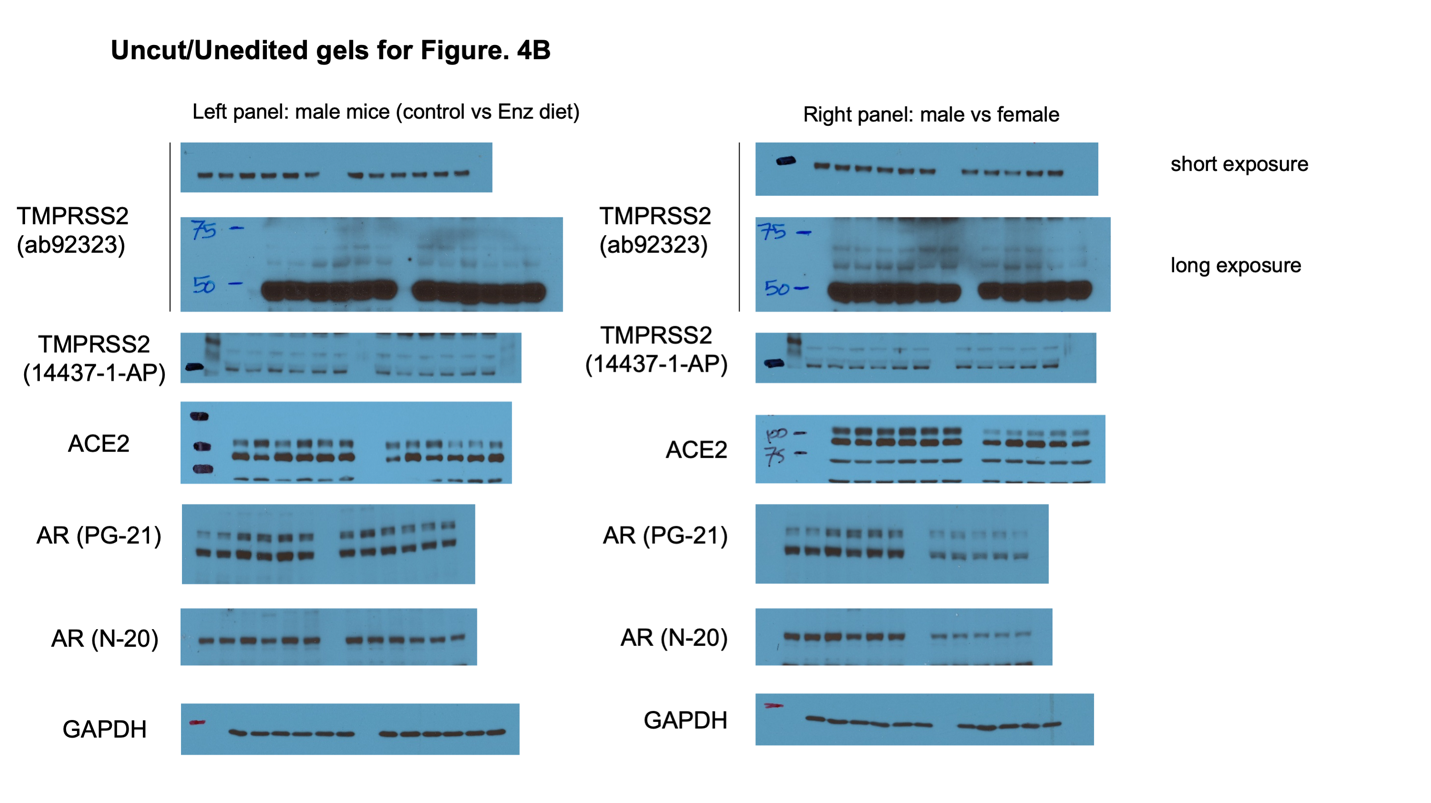

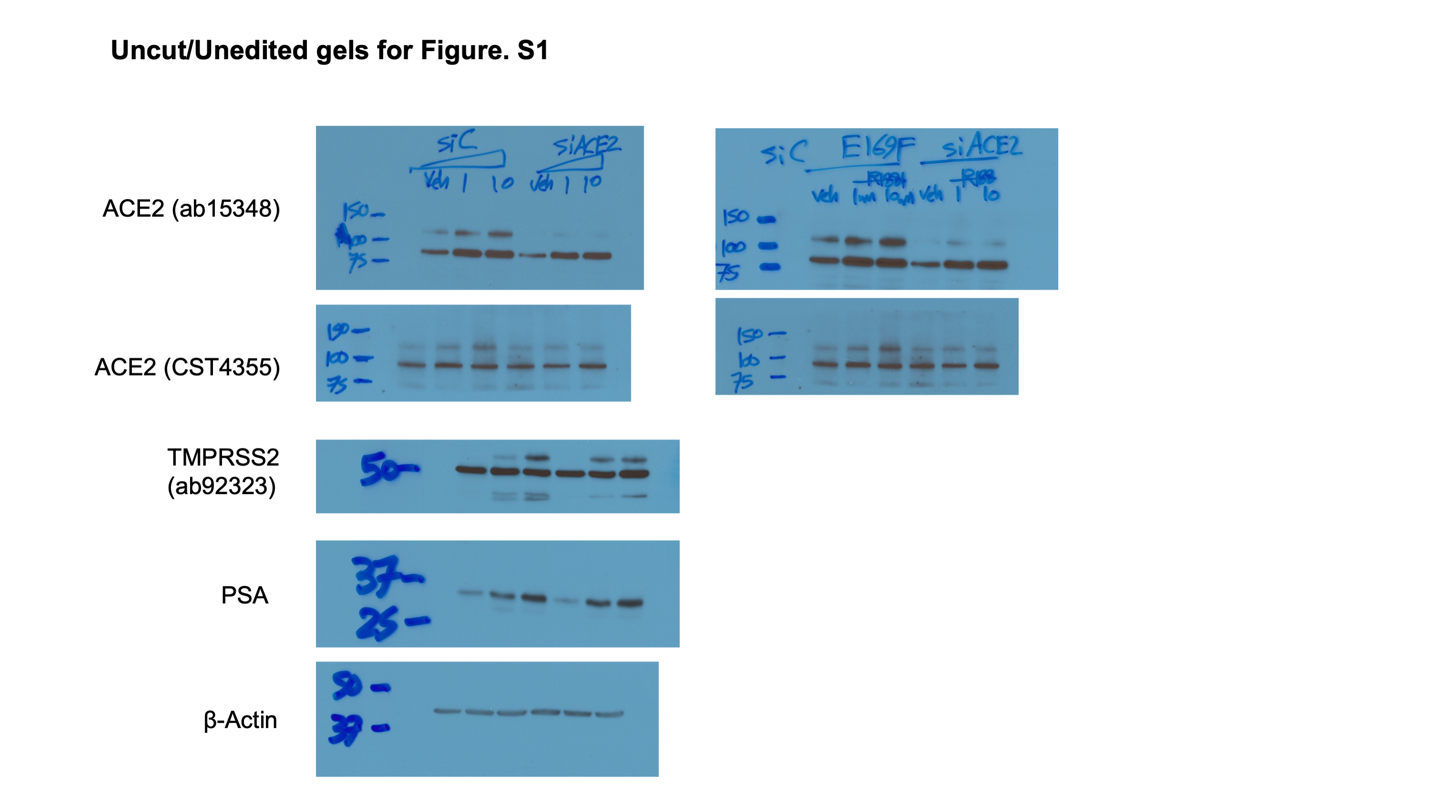

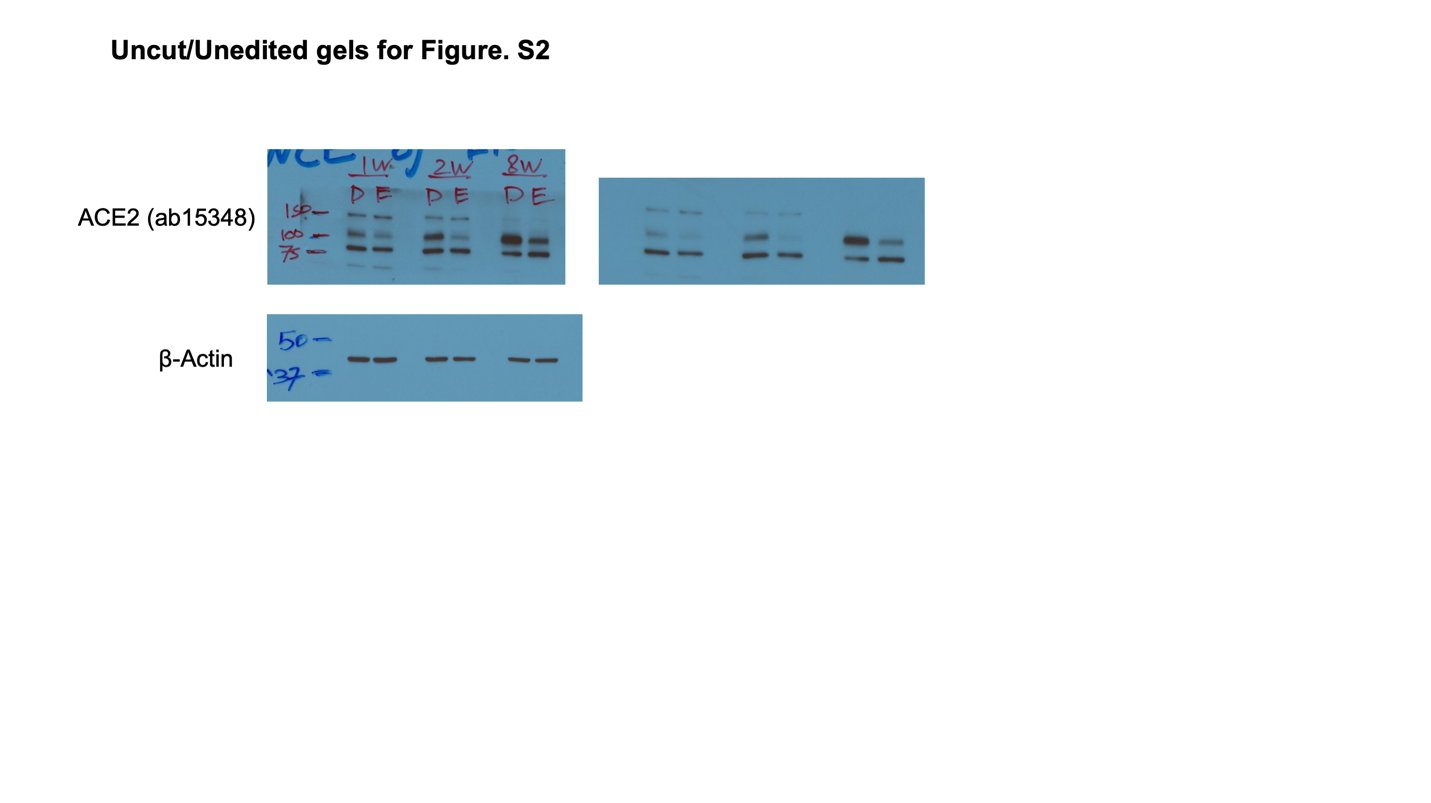

Supplement: Supplementary file 1 — Supplementary Information. [file 41598_2021_90491_MOESM1_ESM.docx]
